# SPON2 Is Upregulated through Notch Signaling Pathway and Promotes Tumor Progression in Gastric Cancer

**DOI:** 10.3390/cancers12061439

**Published:** 2020-06-01

**Authors:** Hyeon-Gu Kang, Won-Jin Kim, Myung-Giun Noh, Kyung-Hee Chun, Seok-Jun Kim

**Affiliations:** 1 Department of Biomedical Science, Department of Life Science & BK21-Plus Research Team for Bioactive Control Technology, College of Natural Sciences, Chosun University, 309 Pilmun-daero, Dong-gu, Gwangju 61452, Korea; kang84562@nate.com (H.-G.K.); wjsh003@naver.com (W.-J.K.); 2 Department of Biomedical Science and Engineering, Gwangju Institute of Science and Technology, 123 Cheomdangwagi-ro, Buk-gu, Gwangju 61005, Korea; md.mgnoh@gmail.com; 3 Department of Biochemistry & Molecular Biology, Brain Korea 21 Plus Project for Medical Science, Yonsei University College of Medicine, 50 Yonsei-ro, Seodaemun-gu, Seoul 03722, Korea

**Keywords:** gastric cancer, spondin-2 (SPON2), notch signaling, cancer progression

## Abstract

Spondin-2 (SPON2) is involved in cancer progression and metastasis of many tumors; however, its role and underlying mechanism in gastric cancer are still obscure. In this study, we investigated the role of SPON2 and related signaling pathway in gastric cancer progression and metastasis. SPON2 expression levels were found to be upregulated in gastric cancer cell lines and patient tissues compared to normal gastric epithelial cells and normal controls. Furthermore, SPON2 silencing was observed to decrease cell proliferation and motility and reduce tumor growth in xenograft mice. Conversely, SPON2 overexpression was found to increase cell proliferation and motility. Subsequently, we focused on regulatory mechanism of SPON2 in gastric cancer. cDNA microarray and in vitro study showed that Notch signaling is significantly correlated to SPON2 expression. Therefore, we confirmed how Notch signaling pathway regulate SPON2 expression using Notch signaling-related transcription factor interaction and reporter gene assay. Additionally, activation of Notch signaling was observed to increase cell proliferation, migration, and invasion through SPON2 expression. Our study demonstrated that Notch signaling-mediated SPON2 upregulation is associated with aggressive progression of gastric cancer. In conclusion, we suggest upregulated SPON2 via Notch signaling as a potential target gene to inhibit gastric cancer progression.

## 1. Introduction

Gastric cancer is the fifth most common type of cancer and second leading cause of cancer-related deaths worldwide [1,2,3]. Despite current developments in diagnosis as well as surgical and pharmacological approaches, metastasized gastric cancer has been observed to lead to poor prognosis and mortality [4,5,6,7,8]. Often, patients who present most favorable characteristics and undergo curative surgical resection have been reported to die from metastatic recurrence [9]. Although many recent studies have focused on gastric metastasis [10,11], the molecular mechanism underlying this phenomenon has not been completely elucidated [11]. Therefore, we aimed to understand the underlying processes involved in gastric metastasis and focused on specific target genes to improve gastric cancer-related prognosis.

Spondin-2 (SPON2, also known as Mindin, DIL1, or M-Spondin) is an extracellular matrix protein, which is known to bind to integrin receptors [12]. Initially, SPON2 was reported as a diagnostic marker specific for prostate cancer [13,14]. However, recent studies have shown that SPON2 is upregulated in various carcinomas including colorectal carcinoma, hepatocellular carcinoma, laryngeal squamous cell carcinoma, and gastric cancer, as well as prostate cancer [15,16,17,18]. SPON2 has been reported to regulate M1-like macrophage recruitment and hippo pathway and plays role in endothelial to mesenchymal transition [19]. In addition, colorectal cancer has been shown to be upregulated by transcription factors such as metastasis-associated in colon cancer protein 1 (MACC1) or early growth response protein 1 (EGR1), which are involved in angiogenesis and metastasis [20,21].

Thus, SPON2 has been reported to be differentially expressed in various carcinomas and contributes to poor prognosis of diverse cancers including gastric cancer [18]. However, the mechanism regulating SPON2 expression needs to be investigated as its role in gastric cancer remains obscure. To understand the regulatory mechanism of SPON2, we inserted *SPON2* transcriptional regulatory motif into luciferase reporter plasmid to confirm the promoter activity. We found the position of the active motif and assumed that recombining binding protein suppressor of hairless (RBP-Jk, CBF-1, or CSL), a Notch signaling-related transcription factor, might be binding to the motif, which was identified through a transcription factor prediction program.

Notch signaling has been reported to be highly expressed and activated in gastric cancer [22]. Additionally, Notch signaling has been shown to be significantly correlated with SPON2. Notch signaling is a cascade that has been reported to play a key role in developmental processes, homeostasis, and cell differentiation [23]. In mammals, there are four receptors (Notch1-4) and five ligands (Delta-like ligand-1,-3,-4 and Jagged-1,2) that have been associated to Notch signaling [24]. Both receptors as well as ligands are transmembrane proteins, and signal transduction is known to induce due to cell-to-cell interaction of the signal modules [25]. The terminal point of the cascade is expression of the target gene. Expression of target genes have been reported to show a malignant phenotype that are related to cancer progression, such as tumor development, metastasis, angiogenesis, and epithelial to mesenchymal transition [26].

In this study, we aimed to elucidate the role of SPON2 in gastric cancer progression. Additionally, we revealed that SPON2 expression is regulated by Notch signaling pathway. This is an extended evidence that Notch signaling regulates SPON2 expression to induce gastric cancer metastasis. Our results provide new insights into the role of SPON2 as the target gene of Notch signaling in gastric cancer progression and suggest SPON2 as a potential targeting molecule in gastric cancer therapy or as a biomarker for prognosis and diagnosis.

## 2. Results

### 2.1. SPON2 Is Upregulated in Patients with Gastric Cancer and Influences Cancer Progression

**Figure 1:**
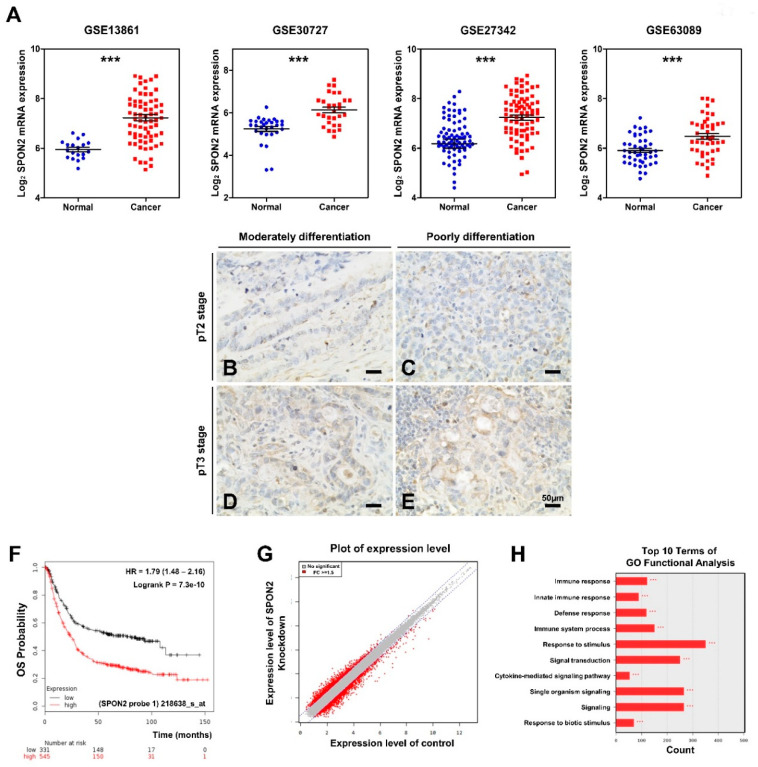
Spondin-2 upregulates in patients with gastric cancer and is associated with poor prognosis. (**A**) Detection of spondin-2 (*SPON2*) mRNA expression levels in patients with gastric cancer using Gene Expression Omnibus (GEO) database. Four datasets were used: GSE13861 (*n* = 90), GSE30727 (*n* = 60), GSE27342 (*n* = 160), and GSE63089 (*n* = 90). (**B**–**E**) Representative photomicrographs showing SPON2 expression in gastric cancer tissue microarray (TMA) (*n* = 72). SPON2 expression was observed to be either very weak/no expression (**B** and **C**), or more than moderate expression (**D** and **E**) in tumor cells. (×400 magnification). (**F**) Kaplan–Meier overall survival (OS) plot analysis of public database indicates an association between SPON2 expression level and poor survival rates of patients with gastric cancer. (**G**,**H**) Gene expressions were analyzed by cDNA microarray in SPON2-silenced MKN28 cells. (**G**) Plot shows differentially expressed genes (red dot = FC > 1.5, grey dot = FC < 1.5). (**H**) GO terms of biological process related to genes for FC > 1.5. *p*-values were calculated using Student’s *t*-test, and significant differences are indicated by * (*** *p* < 0.001).

To study the role of SPON2 in gastric cancer, we confirmed SPON2 expression level in dataset of patients with gastric cancer that was publicly available on Gene Expression Omnibus (GEO) database (Figure 1A). *SPON2* mRNA expression was found to be significantly upregulated in tissues of patients with gastric cancer compared to healthy tissues as illustrated by GSE13861, GSE30727, GSE27342, and GSE63089 datasets (Figure 1A). Furthermore, to investigate SPON2 expression in human gastric cancer tissues, we performed immunohistochemical staining on commercialized tissue microarrays (TMAs) (Figure 1B–E, Figure S1, and Table S1). SPON2 expression was found to be significant in tissues with advanced stage of tumor invasion. Moreover, Table S1 indicates an increasing trend of SPON2 expression in poorly differentiated tumor tissues compared to moderately to well differentiated tumor tissues (*p* = 0.067). Next, the Kaplan–Meier plotter (kmplot.com/analysis) was used to generate survival curves from data of patients with gastric cancer (Figure 1F and Figure S2). Overall survival (OS) rate over five years was found to be poor in high SPON2-expressing groups (*n* = 545) compared to low SPON2-expressing groups (*n* = 331) (Figure 1F). Moreover, the Kaplan–Meier analysis revealed that high expression levels of SPON2 and low survival rate were associated with variables of progression-free survival (PFS) and post-progression survival (PPS) of the whole population (Figure S2). Subsequently, we performed cDNA microarray experiments in MKN28 cells (Figure 1G,H). Results showed that diverse gene expressions were changed on *SPON2* knockdown. Furthermore, gene ontology (GO) analysis revealed that SPON2-related genes were enriched in terms associated to signal transduction, immune response, innate immune response, and defense response. These functions were observed to be related to disease development and gastric cancer tumorigenesis [27,28,29]. Thus, these results strongly supported that SPON2 can serve as a valuable predictive factor for patients with gastric cancer.

### 2.2. SPON2 Knockdown Decreases Proliferation and Motility Abilities of Gastric Cancer Cells

**Figure 2:**
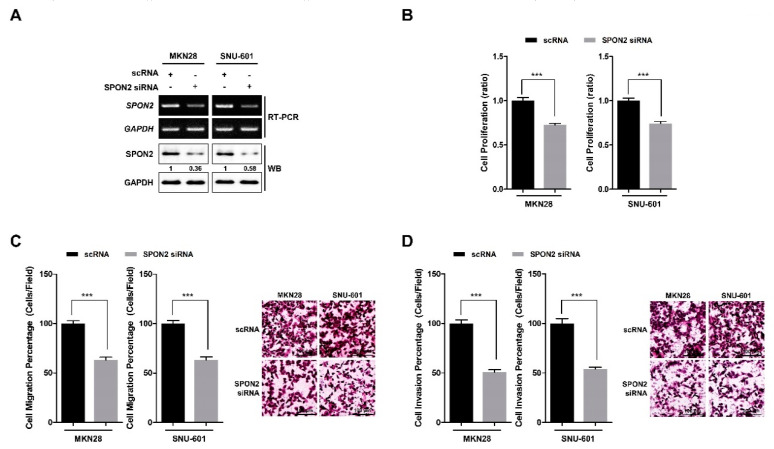
SPON2 downregulation decreases cell proliferation, migration, and invasion abilities of gastric cancer cells. (**A**) MKN28 and SNU-601 cell lines were transfected with small interfering RNA (siRNA) specific for *SPON2* or scrambled siRNA (scRNA). SPON2 expression levels were detected by RT-PCR and Western blotting. GAPDH was used as loading control. (**B**) After 48 h following transfection, water soluble tetrazolium salt-1 (WST-1) assays were performed to detect cell proliferation. (**C**,**D**) Trans-well cell migration and invasion assays were performed to assess (**C**) migration and (**D**) invasion abilities of MKN28 and SNU-601 cells on scRNA or *SPON2* siRNA transfection, which are presented in form of graphs (left) and images (right). Data are presented as mean ± standard error of the mean (SEM) (*n* = 5). *p*-values were calculated using Student’s *t*-test, and significant differences are indicated by * (*** *p* < 0.001).

SPON2 expression level was confirmed by reverse transcription polymerase chain reaction (RT-PCR) in normal gastric epithelial cell line and seven gastric cancer cell lines (Figure S3). Data showed that the majority of the gastric cancer cell lines had high SPON2 expression levels compared to GES-1 normal gastric epithelial cells. Among gastric cancer cell lines, we selected AGS and SNU-668 cells (having relatively low SPON2 expression) and MKN28 and SNU-601 cells (having relatively high SPON2 expression) for subsequent experiments. Further, SPON2 expression was silenced using two specific siRNAs, and the interference efficiency was confirmed by RT-PCR and Western blot analyses (Figure 2A and Figure S4A). *SPON2* knockdown was found to inhibit cell proliferation in MKN28 and SNU-601 cells (Figure 2B and Figure S4B). Moreover, knockdown of SPON2 was observed to inhibit the migratory and invasive abilities of gastric cancer cells as revealed by trans-well cell migration and invasion assay (Figure 2C,D and Figure S4C,D). Therefore, these results showed that silencing SPON2 expression decreases proliferation and cell motility of gastric cancer cells.

### 2.3. SPON2 Overexpression Increases Proliferation and Motility Abilities of Gastric Cancer Cells

**Figure 3:**
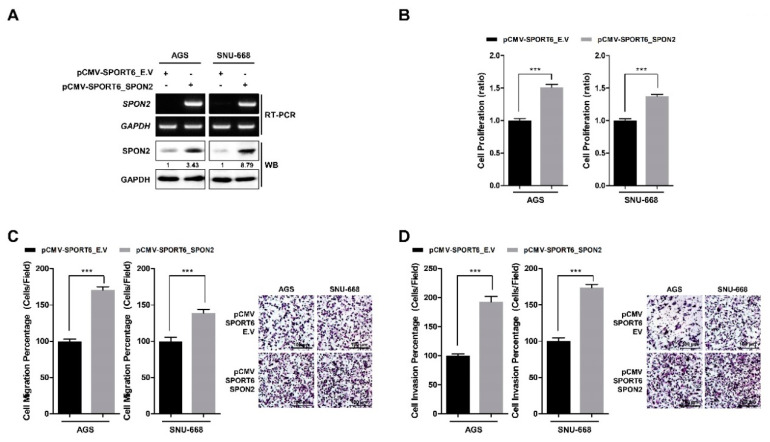
SPON2 overexpression promotes cell proliferation, migration, and invasion in gastric cancer cells. (**A**) Overexpression of SPON2 in AGS and SNU-668 cells was achieved by transfection with an empty vector (pCMV-SPORT6_E.V) or *SPON2*-overexpressing vector (pCMV-SPORT6_SPON2). SPON2 expression levels were detected by RT-PCR and Western blot analysis. (**B**) Cell proliferation was detected by WST-1 assay. (**C**) Cell migration and (**D**) invasion abilities on scRNA or *SPON2* siRNA transfection were detected by trans-well cell migration and invasion assays, which are presented in the form of graphs (left) and images (right). Data are presented as mean ± SEM (*n* = 5). *p*-values were calculated using Student’s *t*-test, and significant differences are indicated by * (*** *p* < 0.001).

Further, we observed the effect of SPON2 overexpression on gastric cancer cell proliferation and cell motility. AGS and SNU-668 cell lines with low endogenous SPON2 expression were transiently transfected with *SPON2*-overexpressing vector (pCMV-SPORT6_SPON2) or empty vector (pCMV-SPORT6_E.V) for 48 h. Efficiency of SPON2 overexpression was confirmed by RT-PCR and Western blot analysis (Figure 3A). Overexpressed SPON2 was observed to significantly upregulate cell proliferation of ASG and SNU-668 cells (Figure 3B). Moreover, cell migration and invasion abilities of ASG and SNU-668 cells were observed to increase (Figure 3C,D). Results showed that overexpression of SPON2 promotes cell proliferation, migration, and invasion in gastric cancer cells. Furthermore, analysis of Figure 2 and Figure 3 indicated that SPON2 regulates gastric cancer cell proliferation and cell motility.

### 2.4. In Vivo Effect of SPON2 Inhibition on Gastric Cancer in Xenograft Mouse Model

**Figure 4:**
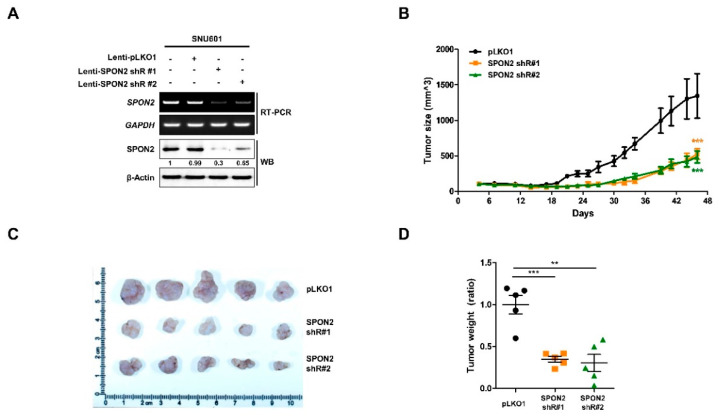
Effect of SPON2 inhibition on tumor growth in xenograft mice. (**A**) Stable *SPON2*-silenced SNU-601 cells were generated by lentivirus infection containing *SPON2*-specific shRNA #1 or shRNA #2. Lentivirus expressing pLKO1_E.V was used as control. Following infection, decreased SPON2 expression levels were detected by RT-PCR and Western blot analysis. (**B**) Stable *SPON2*-silenced cells (50 µL, 2 × 10^6^ cells) with matrigel were implanted in Balb/c nude mice to generate xenografted mice model (each group of pLKO1, SPON2 shR#1, and SPON2 shR#2, *n* = 5). Tumor sizes were measured three times per week until sacrificed at day 47. (**C**,**D**) Xenograft tumors were isolated from the sacrificed mice at day 47. (**C**) Resected tumor was fixed with formaldehyde and visualized using documented immunostaining method. (**D**) 5 Tumors of each group were quantified using microbalance and histogram were developed. *p*-values were calculated using Student’s *t*-test, and significant differences are indicated by * (** *p* < 0.01, *** *p* < 0.001).

We investigated the effect of SPON2 inhibition on tumorigenic ability in xenograft mouse model. We generated two stable *SPON2*-silenced SNU-601 cell lines using lentivirus vector including *SPON2* shRNA #1 or *SPON2* shRNA #2 and evaluated cell proliferation (Figure 4A and Figure S5). Cell lines with validated shRNAs that effectively depleted SPON2 expression on transfection, were also observed to have reduced cell proliferation. Subsequently, these cell lines were injected subcutaneously in nude mice (Figure 4B). Compared to tumors derived from negative control pLKO 0.1-expressing SNU601 cells, *SPON2* knockdown group (*SPON2* shRNA #1 and *SPON2* shRNA #2) of mice showed reduced tumor growth rate at post-injection day 18. Mice were sacrificed before tumor size reached 1500 mm^3^, and mice tumor burdens were separated and visualized (Figure 4C). Additionally, tumor burden weight of *SPON2* knockdown group was found to be significantly reduced compared to the control group (Figure 4D). These data strongly suggested that *SPON2* knockdown reduces gastric cancer progression in vivo.

### 2.5. SPON2 Expression Is Regulated by Notch Signaling Pathway

**Figure 5:**
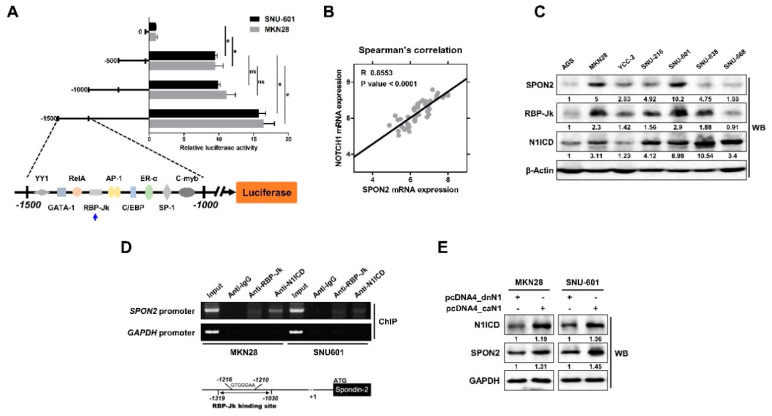
SPON2 expression is regulated by Notch signaling pathway. (**A**) MKN28 and SNU-601 cells were transfected with pGL3-luciferase vector containing *SPON2* promoter binding motifs. Activity was confirmed at motifs extending from position −500 bp by luciferase reporter assays. Subsequently, the region where Notch signaling-related transcriptional factor was expected to bind was identified. The predicted transcription factor was confirmed using promo3.0 (http://alggen.lsi.upc.es). (**B**) Spearman’s correlation analysis indicates the relationship between SPON2 and Notch1 in patients with gastric cancer based on data obtained from publicly available datasets such as GSE63089 (*n* = 45). (**C**) Expression levels of SPON2, recombining binding protein suppressor of hairless (RBP-Jk), and Notch1 intracellular domain (N1ICD) in seven human gastric cancer cell lines as evaluated by Western blot analysis. (**D**) Chromatin immunoprecipitation (ChIP) assay was performed using anti-RBP-Jk and anti-N1ICD, which detected interacting DNA between −1319 bp to −1030 bp. Total genomic DNA in the input lane was used as PCR control. GAPDH promoter region was used as negative control for non-specific binding. (**E**) MKN28 and SNU-601 cells were transfected with pcDNA4_constitutively active Notch1 (caN1) or pcDNA4_dominant-negative Notch1 (dnN1). pcDNA4_dnN1 was used as dominant negative control for pcDNA4_caN1. Expression levels of N1ICD and SPON2 were detected by Western blot analysis.

From the above data, we demonstrated the oncogenic role of SPON2 in gastric cancer progression. Therefore, we further focused on the mechanism by which SPON2 expression is regulated. At first, we inserted a promoter binding region, which was combined with *SPON2* transcriptional motif, in the luciferase reporter vector. Transcriptional activity at motifs up to −1500 bp was then confirmed by luciferase assay (Figure 5A). Results showed a significant increase between 0 bp and −500 bp or between −1000 bp and −1500 bp. We assumed that there must be various transcriptional regulatory motifs in the region between 0 bp and −500 bp. However, it was difficult to segregate the significance, and thus, we focused on the activity between −1000 bp and −1500 bp. Transcriptional factors that are expected to bind to sequences between −1000 bp and −1500 bp were investigated using prediction program (Figure 5A). From the binding motif, we identified the binding site for RBP-Jk, a Notch signaling-related transcription factor. RBP-Jk is a DNA binding protein that interacts with NICD and is involved in transcriptional regulation. Moreover, previously published data suggested the correlation between Notch1 and SPON2 expression (Figure 5B). Data obtained in this study with Spearman’s correlation (R = 0.8553) showed that there is a strong correlation between Notch1 and SPON2. Additionally, we confirmed the basal expression level of SPON2, RBP-Jk, and N1ICD in seven gastric cancer cell lines. These data showed that SPON2 expression was related to Notch signaling (Figure 5C). Moreover, we confirmed that RBP-Jk binding motif is present in the (−1319–−1030 bp) promoter region of SPON2 using chromatin immunoprecipitation (ChIP) assay (Figure 5D). Subsequently, we confirmed the data by transfecting constitutively active (ca) Notch1 plasmid vector (pcDNA4_caN1) in MKN28 and SNU-601 cells. Overexpression of caN1 showed increased transcriptional activity compared to the dominant negative (dn) control (pcDNA4_dnN1) (Figure S6). In addition, Western blot analysis revealed increased SPON2 expression levels (Figure 5E). These results suggested that enhanced Notch signaling increases SPON2 expression via its transcriptional regulation.

### 2.6. Induced SPON2 via Notch Signaling Pathway Promotes Cell Proliferation, Migration, and Invasion in Gastric Cancer Cells

**Figure 6:**
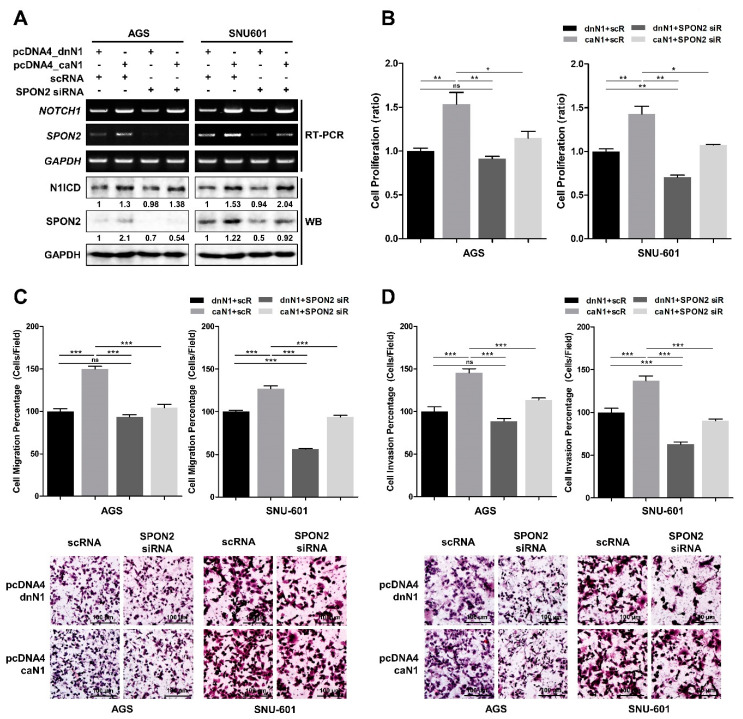
Activated Notch1 enhances gastric cancer cell proliferation and motility through upregulation of SPON2. AGS and SNU-601 cells were co-transfected with pcDNA4_caN1 or pcDNA4_dnN1 vector, and *SPON2* siRNA or scRNA. (**A**) mRNA and protein expression levels of N1ICD and SPON2 were detected by RT-PCR and Western blot analyses, respectively. (**B**) Cell proliferation assay was performed. Results were measured by WST-1 assay after 48 h following transfection. (**C**) Cell migration (**D**) and invasion abilities of AGS and SNU-601 cells on scRNA or *SPON2* siRNA, and pcDNA4_dnN1 or pcDNA4_caN1 transfection were detected by trans-well cell migration and invasion assays, which are presented in the form of graphs (up) and images (down). Data are presented as mean ± SEM (*n* = 5). *p*-values were calculated using Student’s *t*-test, and significant differences are indicated by * (* *p* < 0.05, ** *p* < 0.01, *** *p* < 0.001).

Subsequently, we were interested whether Notch signaling pathway can regulate cell motility and invasion through SPON2 expression. Accordingly, we overexpressed pcDNA4_caN1 and pcDNA4_dnN1 in AGS and SNU-601 cells, and later, we reduced SPON2 expression using *SPON2* siRNA in these cells (Figure 6). The expression levels of N1ICD and SPON2 were evaluated by RT-PCR and Western blot analysis (Figure 6A). N1ICD overexpression was observed to upregulate SPON2 expression in AGS cells and SNU-601 cells. Furthermore, additional treatment with *SPON2* siRNA was observed to stabilize this response. Overexpressed N1ICD also caused enhancement in cell proliferation; however, SPON2 silencing was found to significantly neutralize N1ICD-induced cell proliferation (Figure 6B). In addition, overexpression of N1ICD was observed to increase cell migration and cell invasion (Figure 6C,D). Upregulated cell migration and invasive abilities were found to diminish on *SPON2* silencing, which was similar to that observed with decreased cell proliferation. Furthermore, N1ICD overexpression was observed to promote cell proliferation, migration, and invasion; which was neutralized by *SPON2* siRNA. Therefore, these results suggested that Notch signaling pathway promotes gastric cancer cell proliferation, migration, and invasion via SPON2 upregulation.

### 2.7. Inactivation of Notch Signaling by γ-Secretase Inhibitor Decreases SPON2 Expression Levels in Gastric Cancer Cells

**Figure 7:**
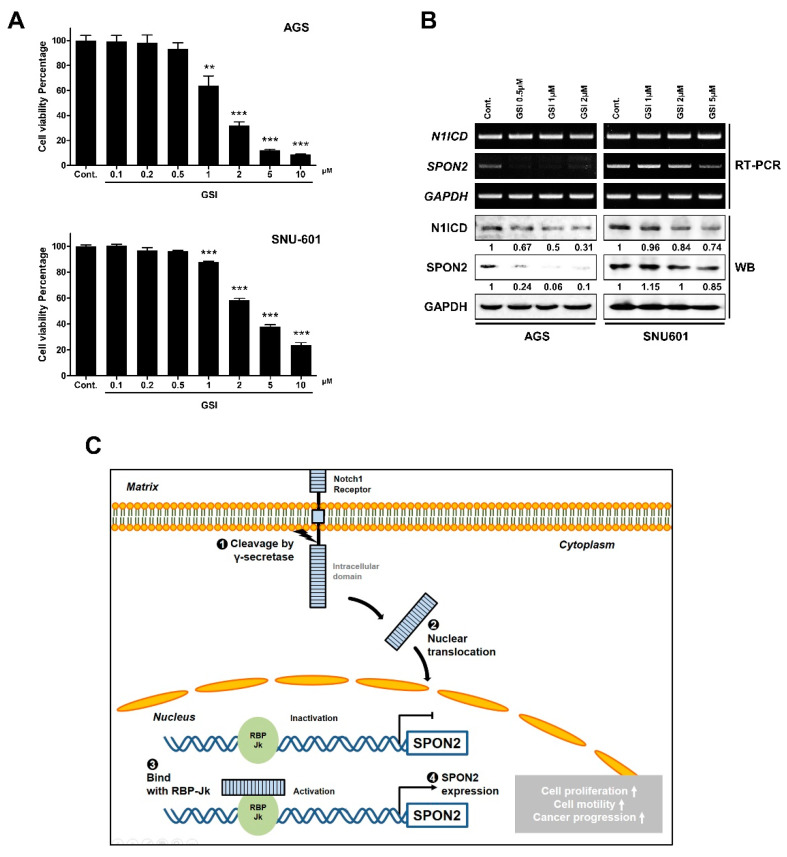
SPON2 expression levels are regulated on inhibiting Notch signaling pathway in AGS and SNU-601 cells. (**A**) Cell viability of AGS and SNU-601 cells after 24 h treatment with γ-secretase inhibitor (GSI) (0.1, 0.2, 0.5, 1, 2, 5, and 10 μM) was detected by WST-1 assay. Control was treated with 0.1% dimethyl sulfoxide (DMSO). Data are presented as mean ± SEM (*n* = 5). *p*-values were calculated using Student’s *t*-test, and significant differences are indicated by * (** *p* < 0.01, *** *p* < 0.001). (**B**) mRNA and protein expression levels of SPON2 and N1ICD in AGS and SNU-601 cells on GSI treatment were detected by RT-PCR and Western blot analysis. (**C**) Schematic model of regulatory mechanism of SPON2 expression through Notch signaling pathway. Activated N1ICD via γ-secretase translocates in nucleus and binds with RBP-Jk in *SPON2* promoter region. Subsequently, SPON2 regulates gastric cancer cell proliferation, migration, and invasion.

Notch signaling can be inactivated using γ-secretase inhibitor (GSI), as it inhibits cleaving of receptor by γ-secretase. Therefore, two gastric cancer cells (AGS and SNU-601) were observed to have decreased Notch signaling on GSI treatment (Figure 7A). γ-secretase works specifically for Notch signaling as well as over 100 substrates such as CD44 and various growth factor receptors [30,31,32]. Therefore, GSI showed high toxicity to the cells. To verify if Notch signaling was inactivated, N1ICD expression levels were evaluated on GSI treatment using RT-PCR and Western blot analysis (Figure 7B). Inactivation of Notch signaling using GSI was found to decrease mRNA and protein expression levels of SPON2. Similarly, transcriptional activity of the SPON2 −1500 bp region was decreased by GSI (Figure S7). Thus, using GSI that inhibits Notch signaling pathway can be used as a strategy to regulate SPON2 expression.

## 3. Discussion

Although many studies continue to comprehend gastric cancer, there is still lack of understanding. Moreover, the incidence and survival rates of patients with gastric cancer are still poorly understood. Therefore, comprehensive research on gastric cancer treatment and efforts to understand the mechanism of gastric cancer progression and metastasis are needed. Through the report articles, Notch have been addressed as strongly signaling involved in the progression and metastasis in gastric cancer [33,34]. However, it is not obvious which mechanism is responsible, and the target gene is also unclear. Therefore, this study suggests the role of SPON2 as a down-stream target of Notch signaling pathway in gastric cancer progression and motility; we also showed the regulation mechanism of Notch signaling and SPON2 in gastric cancer.

Previous reports have indicated the possibility of SPON2 as a potential target to treat gastric cancer [19]. However, the mechanism by which SPON2 plays a role in gastric cancer is unknown. Therefore, we focused on the role of SPON2 in gastric cancer progression and investigated the mechanism of SPON2 expression using in vitro and in vivo experiments, and public databases on patients with gastric cancer.

At first, we investigated the expression level of SPON2 in patients with gastric cancer using publicly available data. We selected three GEO datasets, which showed that SPON2 expression level is increased in patients with gastric cancer. Additionally, Kaplan–Meier plots suggested that high expression of SPON2 is correlated with poor survival rate. The public database showed that SPON2 expression is associated with malignant phenotype of gastric cancer. Thus, we further confirmed SPON2 expression in gastric cancer cell lines and performed a cDNA microarray analysis. Specifically, SPON2 was observed to be related to signal transduction events and response to stimulus regulation and immune mechanisms. GO analysis of the SPON2-related events has been reported to be involved in cancer development [27]. Consistent with patient data, *SPON2* knockdown was observed to significantly reduce cell proliferation, migration, and invasion in MKN28 and SNU-601 cells, whereas *SPON2* overexpression was found to enhance cell proliferation, migration, and invasive abilities of AGS and SNU-668 cells. Moreover, *SPON2* silencing was observed to decrease tumor growth in xenograft mouse models. These results strongly supported the significant role of SPON2 in gastric cancer cell proliferation and cell motility.

Second, whether SPON2 expression is regulated by Notch signaling was investigated we constructed a plasmid that shared promoter region with *SPON2* to study transcriptional regulatory mechanisms of SPON2. Further, we found a promoter region that regulates SPON2 transcription through luciferase activity. This region was confirmed to bind to CBF-1/RBP-Jk using prediction program. Correlation between the expression levels of Notch1 and SPON2 was evident to support the hypothesis. Furthermore, we observed that SPON2 expression is regulated on overexpression of caN1 vector. Above evidence supported that enhanced Notch signaling induces upregulation of SPON2 expression. Specifically, Notch signaling has been reported to express as highly as Wnt signals in gastric cancer [34,35,36]. Moreover, Notch signaling has been shown to be involved in not only developmental and differentiation processes, but also in gastric cancer progression and metastasis [23,26]. However, studies associated with Notch signaling and gastric cancer metastasis are not abundant compared to reports related to Wnt signaling. Therefore, the role of Notch signaling pathway-regulated SPON2 is additional evidence related to metastasis and is important in gastric cancer progression. In addition, SPON2 expression was observed to be regulated by Notch signaling inhibitor (GSI) in gastric cancer (Figure 7C).

Recently, scientific research on SPON2 in gastric cancer has increased [18,37]. In this article, we proposed a hypothesis that correlates SPON2 expression with the progression and prognosis of gastric cancer and its regulatory mechanisms. Furthermore, SPON2 expression may cause a contradictory result in other cancers. Initially, expression of SPON2 by MACC1 regulation was reported to promote colorectal cancer [20]. However, some studies have shown that SPON2 suppresses colon cancer by blocking angiogenesis [21]. SPON2 has also been shown to recruit M1-like macrophage and inhibit hepatocellular carcinoma; however, the expression levels were observed to increase in malignant hepatocellular carcinoma [16,19]. This suggests that there may be a difference in SPON2 expression depending upon race and region, and this needs to be further studied. Consequently, SPON2 may be a potent target for various cancer progression and immune responses. Interestingly, our cDNA microarray data (Figure 1G,H) showed that inhibition of SPON2 regulates a variety of immune response-associated genes.

In fact, the causes of gastric cancer have been reported to vary, which includes gastritis, *H. pylori* infection, dietary factors, genetic factors, and other environmental factors [38,39]. These high-risk factors have been related to abnormal immune response. As a result, SPON2 is expected to play a significant role in gastric cancer development with inflammation and *H. pylori* infection. Therefore, the relationship of SPON2 and immune-related genes within tumor microenvironment in various cancer is further required to be studied.

Overall, this article proposes that SPON2 is a potent target to improve gastric cancer progression. Furthermore, SPON2 in combination with Notch signaling inhibitor can provide a potential therapeutic strategy to target gastric cancer.

## 4. Materials and Methods

### 4.1. Human Gastric Cancer TMA and Immunohistochemistry

TMAs comprising of 72 tissue samples from patients with gastric cancer were purchased from US Biomax, Inc. (BS0102c; Derwood, MD, USA). Immunohistochemistry was performed to analyze SPON2 expression using VECTASTAIN^®^ ABC kit and DAB substrate kit (Vector Laboratories Inc., Burlingame, CA, USA) following the manufacturer’s instruction. Moreover, the method was performed as described previously [40].

### 4.2. Cell Culture and Chemicals Used

For in vitro study, six human gastric cancer cell lines including AGS, MKN28, SNU-216, SNU-601, SNU-638, and SNU-668 were purchased from Korean Cell Line Bank (KCLB, Seoul, Korea). YCC-2, HEK293FT, and human normal gastric epithelial (GES-1) cells were obtained from Yonsei Cancer Center (Seoul, Korea). All cell lines, except HEK293FT cells, were cultured in RPMI 1640 medium (Welgene, Gyeonsan, Korea) containing 10% fetal bovine serum (FBS) (Corning, Woodland, CA, USA) and 1% streptomycin/penicillin (Gibco, Thermo Fisher Scientific, Waltham, MA, USA) at 37 ℃ in a humidified incubator with 5% CO_2_. HEK293FT cells were cultured in high glucose DMEM (Welgene, Gyeongsan, Korea), which was supplemented with 10% FBS and 1% antibiotics. γ-secretase inhibitor, cbz-IL-CHO (GSI-I, Z-LLNle-CHO), was purchased from Calbiochem (Billerica, MA, USA). GSI was dissolved in 10 mM dimethyl sulfoxide. Dissolved GSI (1 µL/mL) solution was added to the medium and serially diluted to adjust the concentration. Cells were treated with GSI for 24 h.

### 4.3. siRNA Transfection and Plasmid Construction

Human *SPON2* siRNA and *SPON2* plasmids were transfected using Lipofectamine RNAiMAX reagent or Lipofectamine 2000 reagent (Invitrogen, Carlsbad, CA, USA) following the manufacturer’s protocol. Two SPON2-specific siRNAs were purchased from Genolution Inc. (Seoul, Korea): siRNA #1: 5′-GCGCAUAGCUCCGACUACUU-3′ and siRNA #2: 5′-GUAACGGGCUGCGCGACUUUU-3′. Human *SPON2* was cloned using pCMV-SPORT6_SPON2. *SPON2* clone was provided by Korea Human Gene Bank (KHGB, Daejeon, Korea). *SPON2* promoter site was cloned into pGL3-Basic vector plasmid within Hind3 and Xho1 restriction enzyme sites, thereby creating the luciferase reporter plasmid construct. Genomic DNA was PCR amplified using primer (Table S2). Genomic DNA was isolated from SNU-601 cells.

### 4.4. RNA Isolation and RT-PCR

Total RNA was isolated from human gastric cancer cell lines using RNAiso reagent (Takara, Shiga, Japan) following the manufacturer’s protocol. cDNA synthesis was performed using reverse transcription system (Toyobo, Osaka, Japan), and PCR was performed using nTaq DNA polymerase (Enzynomics, Daejeon, Korea). Primers used in the reaction are described in Table S2. PCR products were detected by agarose gel electrophoresis.

### 4.5. Western Blot Analysis

Cells were lysed using RIPA buffer (Biosesang Inc, Seongnam, Korea) containing phosphatase and protease inhibitor cocktail (GeneDEPOT, Barker, TX, USA). Protein concentration was measured using Pierce™ 660 nm protein assay reagent (Thermo Fisher Scientific, Waltham, MA, USA). Proteins (20 μg) were separated by SDS-polyacrylamide gel electrophoresis and transferred onto polyvinylidene fluoride membrane (Merck, Kenilworth, NJ, USA). After blocking with 5% skim milk for 1 h, membranes were incubated overnight at 4 ℃ with primary antibodies, which were dissolved in PBST containing 5% BSA. The following primary antibodies were used: anti-Mindin (SPON2) (Santa Cruz Biotechnology, Santa Cruz, CA, USA)], anti-RBP-Jk (Santa Cruz Biotechnology), and anti-activated Notch1 (Abcam, Cambridge, UK), which detected Notch1 intracellular domain (N1ICD). Anti-GAPDH (Bioworld, Dublin, OH, USA) was used as control. Further, membranes were incubated with HRP-conjugated secondary antibody (Bethyl Laboratories, Montgomery, TX, USA) for 90 min followed by detection using ECL kit (Bio-Rad, Hercules, CA, USA) and Supernova-Q1800.

### 4.6. Cell Proliferation Assay

Cell proliferation assays were performed using cell permeable water-soluble tetrazolium salt, WST-1 (4-[3-(4-Iodophenyl)-2-(4-nitrophenyl)-sH-5-tetrazolio]-1,3-benzene disulfonate). AGS, MKN28, SNU-601, and SNU-668 cells were seeded in 96-well plates (4 × 10^3^ cells/well). Following overnight incubation, cells were transfected with siRNA (scRNA or *SPON2* siRNA) and vector plasmid (pCMV-SPORT6_E.V or pCMV-SPORT6_SPON2). WST-1 solution (Daeil Lab Services Co. Ltd., Seoul, Korea) was added to each well after 48 h following transfection. Plates were incubated for an additional 1–2 h and gently shaken before the absorbance was measured at 450 nm.

### 4.7. Trans-well Migration and Invasion Assays

Human gastric cancer cells were transfected with siRNA (scRNA or *SPON2* siRNA) and vector plasmid (pCMV-SPORT6_E.V or pCMV-SPORT6_SPON2). After 24 h transfection, 2 × 10^4^ cells/200 µL FBS-free medium was added to the trans-well upper chamber (Corning Costar, Cambridge, MA, USA) on a filter coated with 0.5 mg/mL collagen type I (BD Biosciences, Seoul, Korea) for migration assay. For the invasion assay, the chamber was coated with Matrigel (1:15) (BD Biosciences). RPMI 1640 medium containing 10% FBS and 1% antibiotics was added to the lower chamber, and plates were incubated for 20 h. Cells that migrated and invaded were visualized after hematoxylin and eosin staining. For quantification, cells were counted from five randomly selected areas in each well using wide-field microscopy. Data were expressed as mean ± standard error of the mean (SEM) from three independent experiments.

### 4.8. In Vivo Growth Study

All animal experiments were approved by Chosun University Institutional Animal Care and Use Committee (CIACUC) and performed in specific pathogen-free facilities following the guidelines of CIACUC (CIACUC2019-A0004). Six-week-old pathogen-free female Balb/c nude mice were purchased from OrientBio (Seungnam, Korea). Mice were subcutaneously injected into both flanks with 2 × 10^6^ stable SNU-601 (3 types: pLKO1 control, *SPON2* shRNA #1, and *SPON2* shRNA #2) cells in each flank. From palpable tumor formation until termination, tumor sizes were measured every 2–3 days using calipers, and tumor volume was calculated using the formula: length × (width)^2^ × 0.5236. Mice were sacrificed in a 7.5% CO_2_ chamber, and tumors were harvested for further analyses.

### 4.9. Luciferase Reporter Assay

Gastric cancer cells were seeded in 6-well plates (5 × 10^4^ cells/well). Following overnight incubation, cells were transfected with 2 μg of pGL3-basic luciferase reporter plasmid and 0.25 μg of β-galactosidase expression plasmid vector. For co-transfection, 1.5 μg of 4× CSL luciferase reporter plasmid and 0.5 μg expression vector (dnN1 or caN1) were added instead of 2 μg of pGL3 luciferase vector. After incubation for 24 h, cells were harvested and a luciferase assay was performed (Promega, Madison, WI, USA) following the manufacturer’s instructions. A β-galactosidase enzyme assay (Promega) was used as a control to evaluate transfection efficiency.

### 4.10. ChIP Assay

MKN28 and SNU-601 cells were cultured in a 150 mm dish (2 × 10^5^ cells). After incubation for 48 h, cells were treated with 1% formaldehyde for cross-linking. ChIP assays were performed using Pierce™ Agarose ChIP kit (Thermo Fisher Scientific, Waltham, MA, USA) following the manufacturer’s protocol. Antibodies used for ChIP—anti-activated Notch1 and anti-RBP-Jk—were equivalent to the antibodies used in Western blot analysis. Anti-rabbit immunoglobulin G (IgG) was purchased from Santa Cruz Biotechnology (CA, USA) and used as negative control. Primers were designed using SPON2 promoter binding sites, and RT-PCR was performed. Primers used in the reaction are described in Table S2. Primer with GAPDH promoter site was obtained from ChIP kit. RT-PCR was performed using Ex Taq DNA polymerase (Takara, Shiga, Japan).

### 4.11. Gene Expression Profile Data and Kaplan-Meier Analysis

The available datasets; GSE13861, GSE30727, GSE27342, and GSE63089 were downloaded from the GEO database (http://www.ncbi.nlm.nih.gov/geo/). The four datasets were normalized using GEO2R, and a scatter plot was obtained for expression pattern analysis. Kaplan–Meier curves for OS, PFS, and PPS of patients with gastric cancer were generated using an online resource, i.e., Kaplan-Meier Plotter (http://kmplot.com/analysis) [41].

### 4.12. Statistical Analysis

Statistical analyses were summarized using GraphPad Prism 5 program (GraphPad Software, Inc., San Diego, CA, USA). Data were analyzed using Student’s *t*-test, unless otherwise specified. Data from public databases were analyzed using the Kaplan–Meier plotter to determine the differences in patient survival. Results were presented as mean ± SEM. *p* < 0.05 was considered to be statistically significant.

## 5. Conclusions

In conclusion, we demonstrated that SPON2 is an important regulator of gastric cancer progression. Furthermore, we show that the expression and functioning of SPON2 is regulated by Notch signaling pathway via RBP-Jk transcription factor. Overall, these results suggest SPON2 as a potential therapeutic target for impeding gastric cancer progression, which can be regulated by Notch signaling inhibition.

## Supplementary Materials

The following are available online at https://www.mdpi.com/2072-6694/12/6/1439/s1, Figure S1: High expression levels of SPON2 in tissue microarray samples with advanced stage of gastric cancer, Figure S2: Survival rate based on SPON2 expression in patients with gastric cancer is illustrated, Figure S3: Detection of basal expression basal level of *SPON2* mRNA in gastric cancer cell lines, Figure S4: SPON2 silencing decreases cell proliferation, migration, and invasion of gastric cancer cells, Figure S5: Effect of SPON2 inhibition on cell proliferation in stable SNU-601 cells, Figure S6: Transcriptional activity of Notch signaling pathway on caN1 overexpression in gastric cancer cells, Figure S7: Effect of GSI on SPON2 transcriptional activity, Table S1: Clinicopathologic features of patients with gastric cancer, Table S2: Primer sequences used in RT-PCR.
