# Supplementary material for: SPON2 Is Upregulated through Notch Signaling Pathway and Promotes Tumor Progression in Gastric Cancer
Source: Cancers (Basel). 2020 Jun 1;12(6):1439. doi: 10.3390/cancers12061439 (PMC7352369; doi:10.3390/cancers12061439)
Supplement: Supplementary file 1 [file cancers-12-01439-s001.zip › cancers_803439_supp_for_XML.pdf]

Supplementary material

SPON2 is Upregulated through Notch Signaling Pathway and Promotes Tumor Progression in Gastric Cancer

Hyeon-Gu Kang [1], Won-Jun Kim [1], Myung-Giun Noh [2], Kyung-Hee Chun [3,*], and Seok-Jun Kim [1,*]

Table 1. Clinicopathologic features of patients with gastric cancer.

**Characteristics**	**Number of patients**	**Low SPON2 expression, number (%)**	**High SPON2 expression, number (%)**	***p***
Age (year)				
<60	32	20 (27.8)	10 (13.9)	0.800
≥60	40	23 (31.9)	15 (20.8)	
Sex				
Male	59	35 (48.6)	22 (30.6)	0.510
Female	13	8 (11.1)	3 (4.2)	
Tumor differentiation				
Moderate–well Differentiation	27	19 (26.4)	6 (8.3)	0.067
Poor differentiation	45	24 (33.3)	19 (26.4)	
Pathologic stage				
pT1–pT2	24	18 (26.5)	4 (5.9)	0.014
pT3–pT4	42	19 (27.9)	21 (30.9)	

Table 2. Primer sequence used in RT-PCR.

SPON2	Sense	5′-GGGAGAGTCCATCTGTTCCG-3′
	Anti-sense	5′-CACAAACGAGACCAGCGAGTG-3′
NOTCH1	Sense	5′-GACGAGTACAACCTGGTGCG-3′
	Anti-sense	5′-AGGTAGCCATGGGGTGACTC-3′
GAPDH	Sense	5′-GGCTGCTTTTAACTCTGGTA-3′
	Anti-sense	5′-ACTTGATTTTGGAGGGATCT-3′
SPON2 ChIP	Sense	5′-GGCACGGGTGTGAGGAGGGG-3′
	Anti-sense	5′-AGTGTCTGGCTGCCTCTCAGG-3′
SPON2 (-1500 bp)	Sense	5′-GATCCTCGAG(***XhoI***)GAGGCCTCTGCTCCCTGCCCTC-3′
SPON2 (-1000 bp)	Sense	5′-GATCCTCGAG(***XhoI***)TCCCACTCAGCTGGCCTCATTG-3′
SPON2 (-500 bp)	Sense	5′-GATCCTCGAG(***XhoI***)CCTCACAGGAGCGCCTCTGGTG-3′
SPON2 (0 bp)	anti-sense	5′-GATCAAGCTT(***HindIII***)TCCGACGACACCGACAAAGGAG-3′

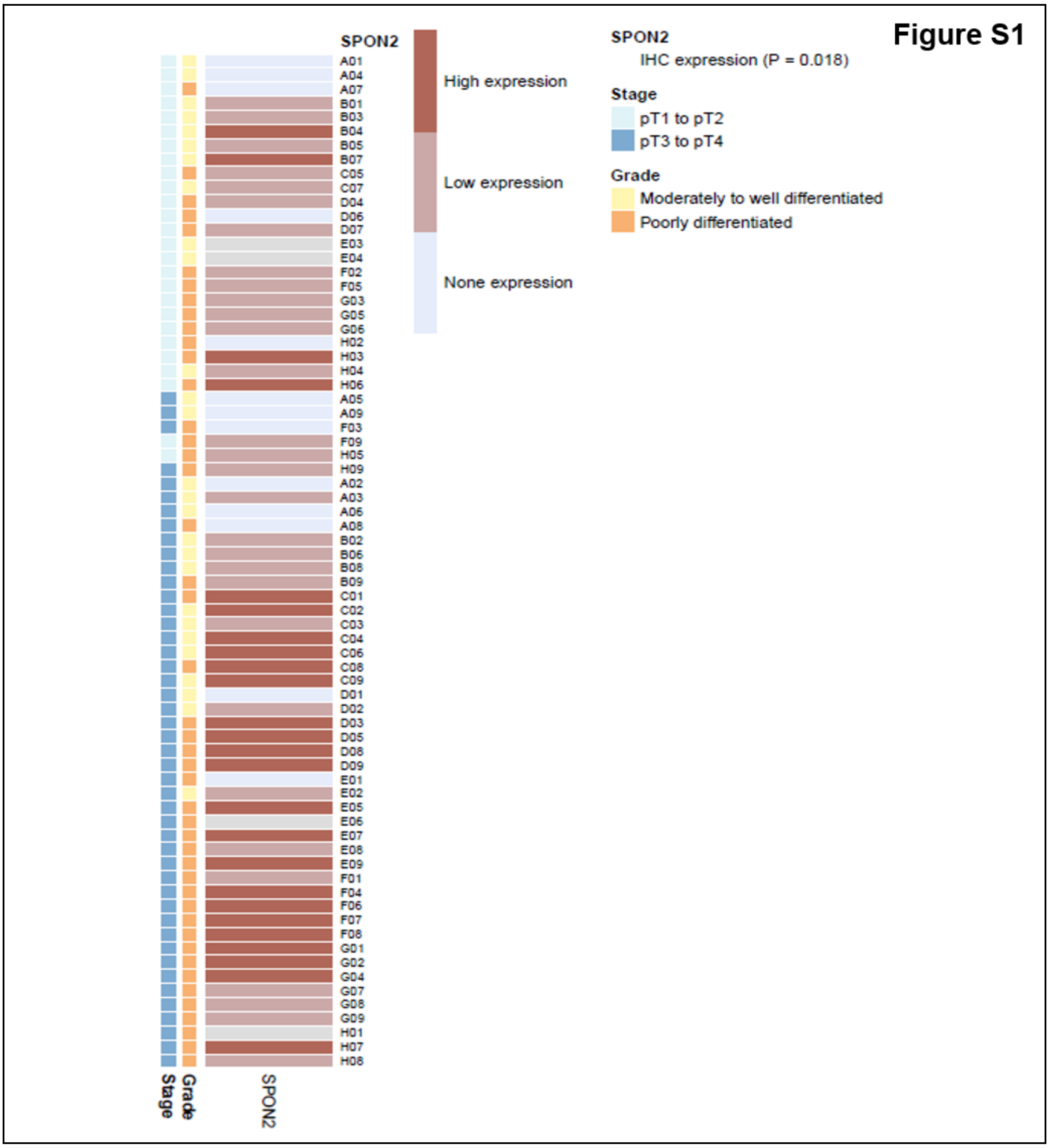

Figure S1. (in support of Fig. 1): High expression levels of SPON2 in tissue microarray samples with advanced stage of gastric cancer. Analysis of SPON2 expression in tumor tissues from 72 patients with gastric cancer based on staining intensity. Staining intensity showed three different expression patterns (none, low, and high) based on related stage and grade.

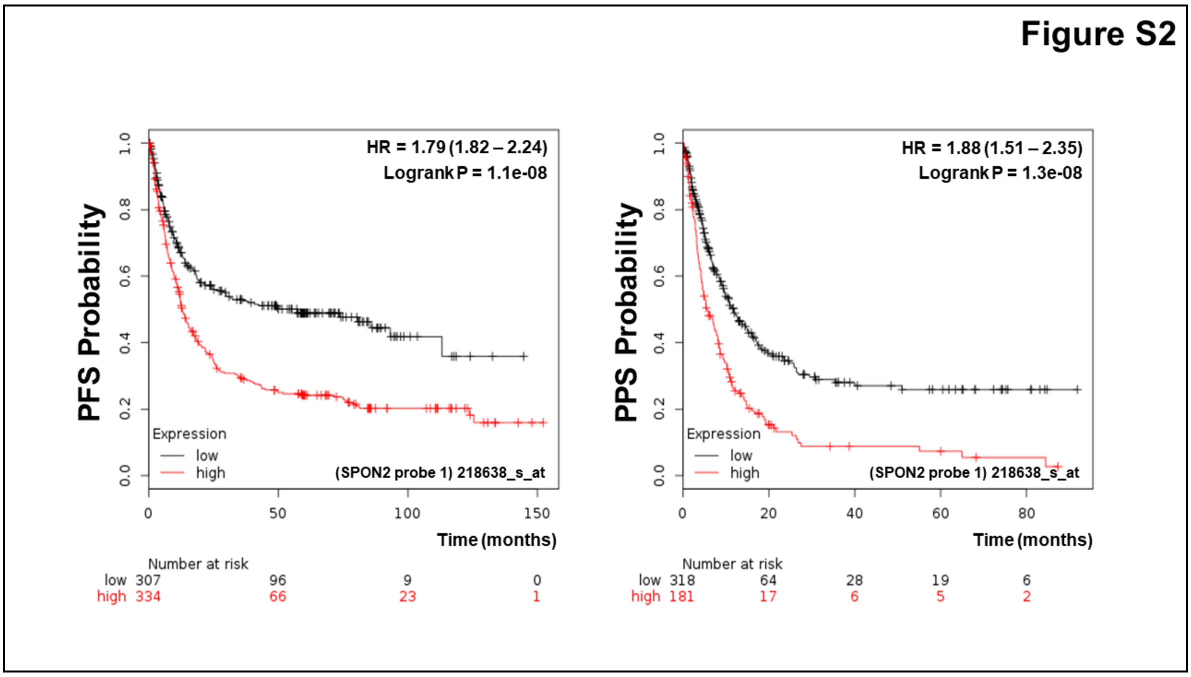

Figure S2. (in support of Fig. 1): Survival rate based on SPON2 expression in patients with gastric cancer. Progression-free survival (PFS, n = 641) and post-progression survival (PPS, n = 499) curves of patients with gastric cancer evaluated using Kaplan-Meier survival analyses.

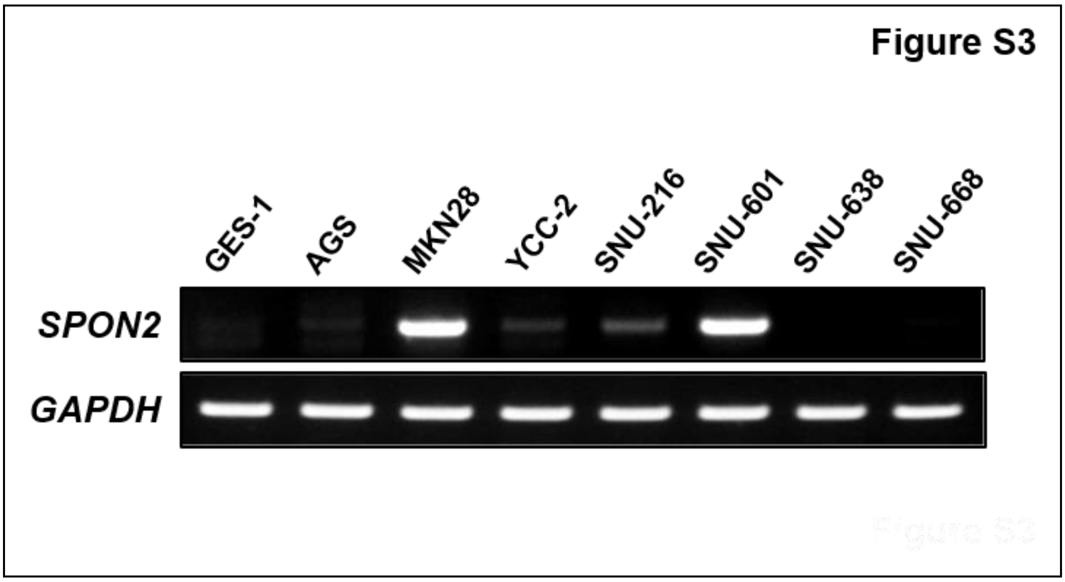

Figure S3. Detection of basal expression level of *SPON2* mRNA in gastric cancer cell lines.*SPON2* mRNA expression levels in normal gastric epithelial cell (GES-1) and seven human gastric cancer cell lines (AGS, MKN28, YCC2, SNU-216, SNU-601, SNU-638, and SNU-668 cells) were evaluated by RT-PCR. GAPDH was used as loading control.

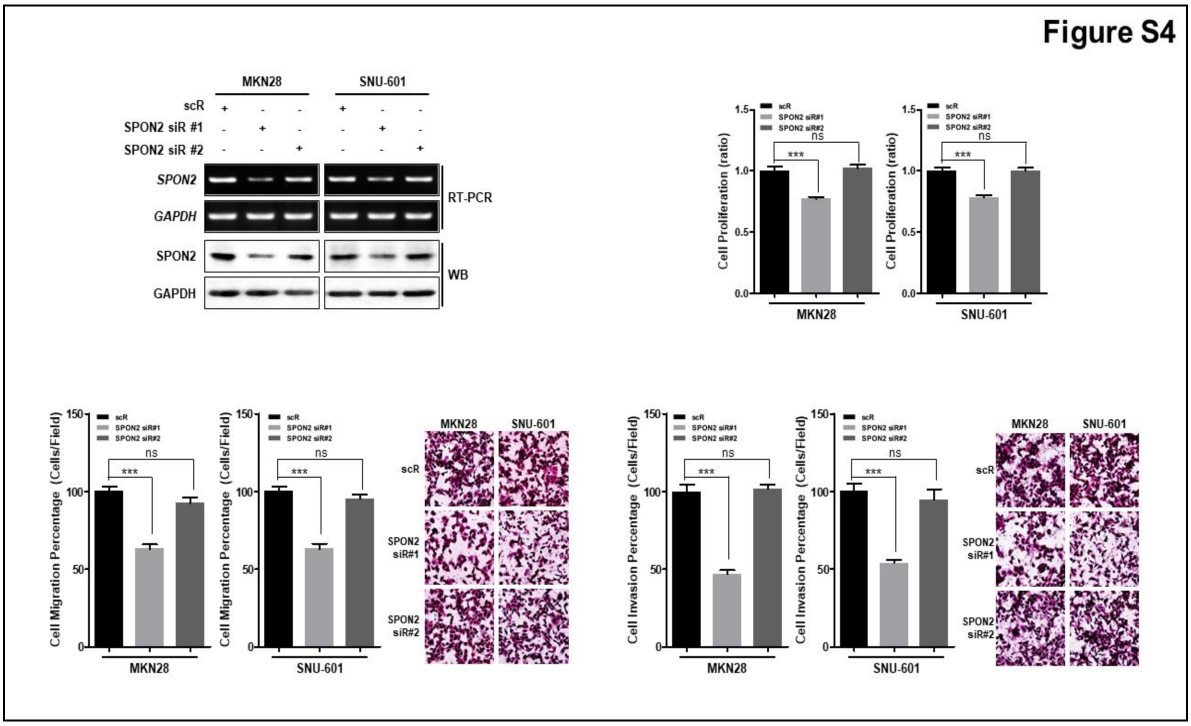

Figure S4 (in support of Fig. 2). SPON2 silencing decreases cell proliferation, migration, and invasion of gastric cancer cells. (A) MKN28 and SNU-601 cell lines were transfected with scrambled siRNA (scRNA) or two *SPON2*-specific siRNAs. SPON2 expression level was detected following transfection with two types of *SPON2* siRNA by RT-PCR and western blot analysis. (B) Cell proliferation, (C) cell migration, and (D) cell invasion were detected in MKN-28 and SNU-601 cells using scRNA or two types of *SPON2* siRNA transfection. Data are presented as mean ± SEM (n = 5). p-values were calculated using Student's t-test and significant differences are indicated by * (*** $p <$ 0.001).

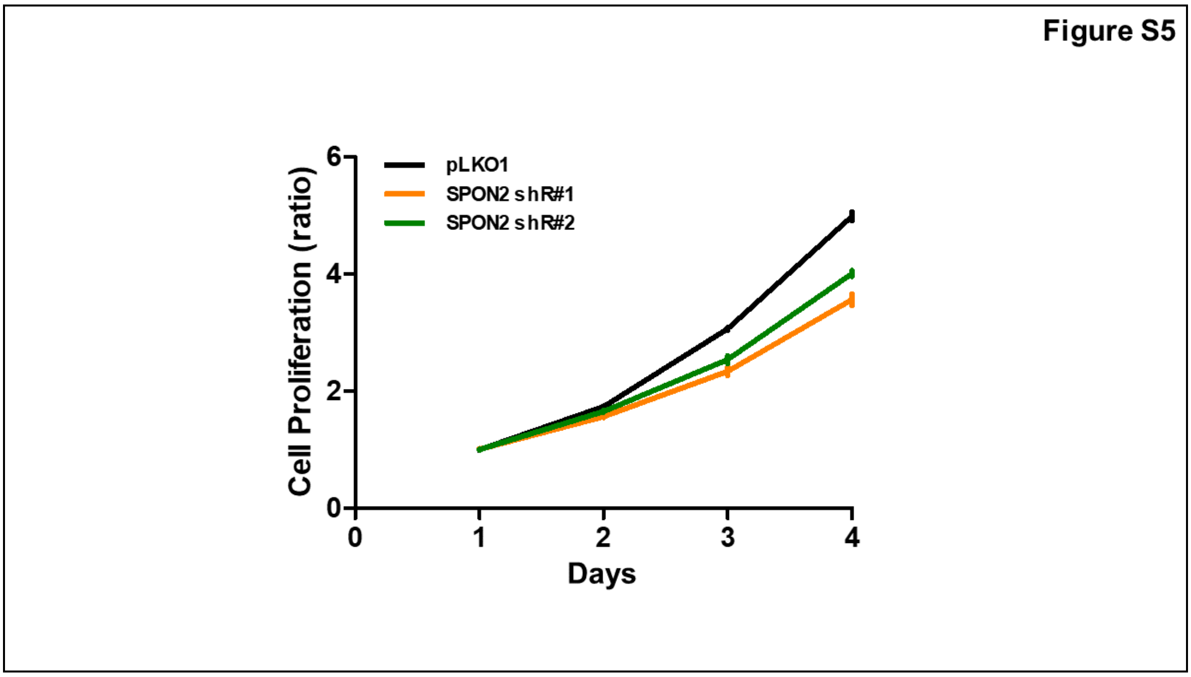

Figure S5 (in support of Fig. 4). Effect of SPON2 inhibition on cell proliferation in stable SNU-601 cells. We generated stable SNU-601 cell lines using lentiviruses expressing *SPON2*-specific shRNA #1 and shRNA #2, and decreased SPON2 expression in these stable cell lines. Data are presented as mean ± SEM (n = 5). p-values were calculated using Student's t-test and significant differences are indicated by * (*** $p < 0.001$).

Figure S6

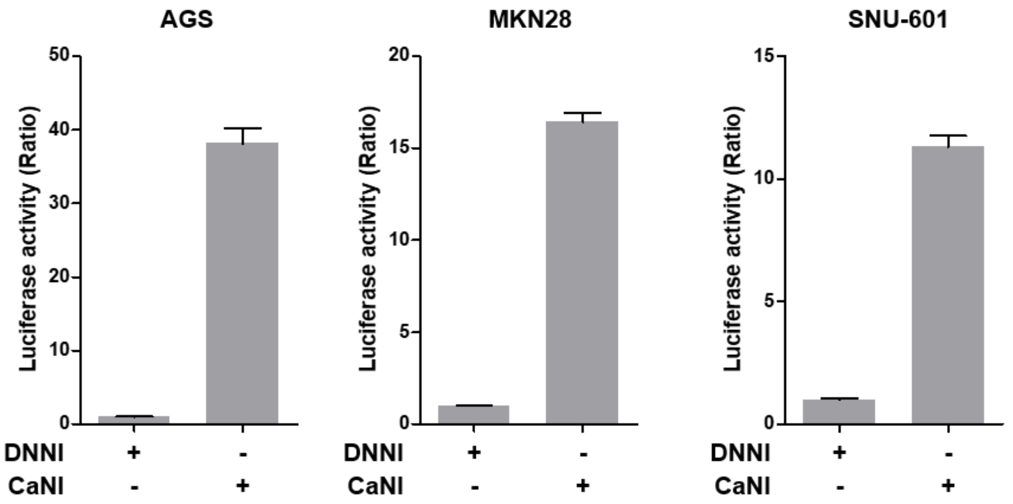

Figure S6 (in support of Fig. 5). Transcriptional activity of Notch signaling pathway on caN1 overexpression in gastric cancer cells. dnN1 and caN1 were transfected with luciferase reporter plasmid containing CSL domain (4× CSL luciferase vector). Transcriptional activity was confirmed through luciferase reporter assay in AGS, MKN28, and SNU-601 cells. Data are presented as mean ± SEM (n = 5). p-values were calculated using Student's t-test and significant differences are indicated by * (*** $p < 0.001$).

Figure S7

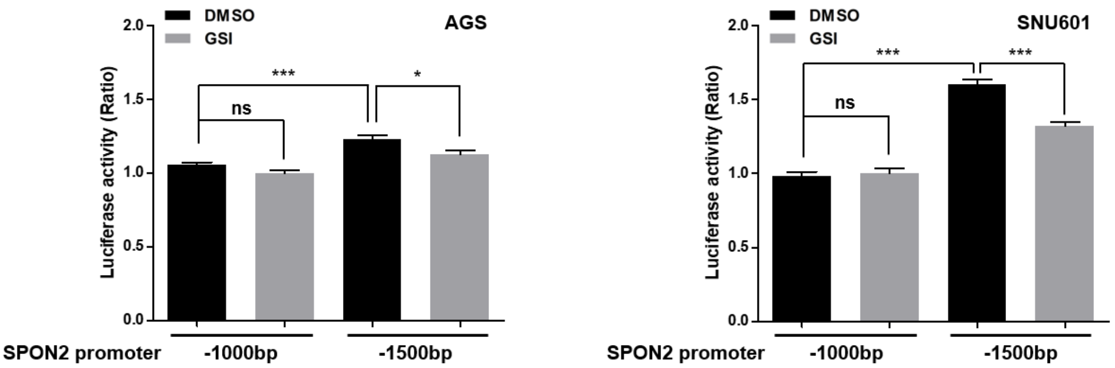

Figure S7 (in support of Figure 7). Effect of GSI on SPON2 transcriptional activity. pGL3-luciferase vector containing SPON2 promoter binding motifs were transfected on AGS and SNU-601 cells. After 24 h, proper concentration of GSIs (AGS 1 μM and SNU-601 2 μM) were treated. The cells were further incubated for 24h and measured through luciferase reporter assay. Data are presented as mean ± SEM (n = 5). p-values were calculated using Student's t-test and significant differences are indicated by * (* $p < 0.05$, *** $p < 0.001$).
