# Supplementary figures and images for: SPON2 Is Upregulated through Notch Signaling Pathway and Promotes Tumor Progression in Gastric Cancer
Source: Cancers (Basel). 2020 Jun 1;12(6):1439. doi: 10.3390/cancers12061439 (PMC7352369; doi:10.3390/cancers12061439)

Fig. 2A

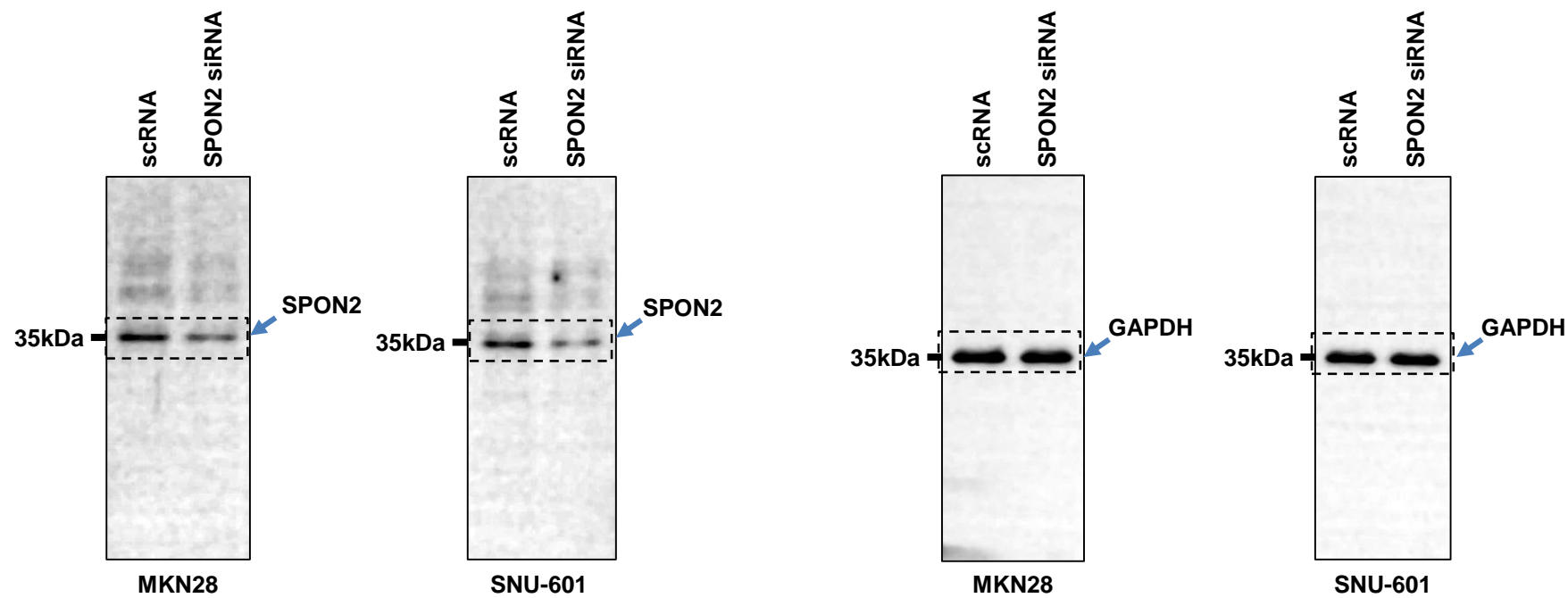

Fig. S3

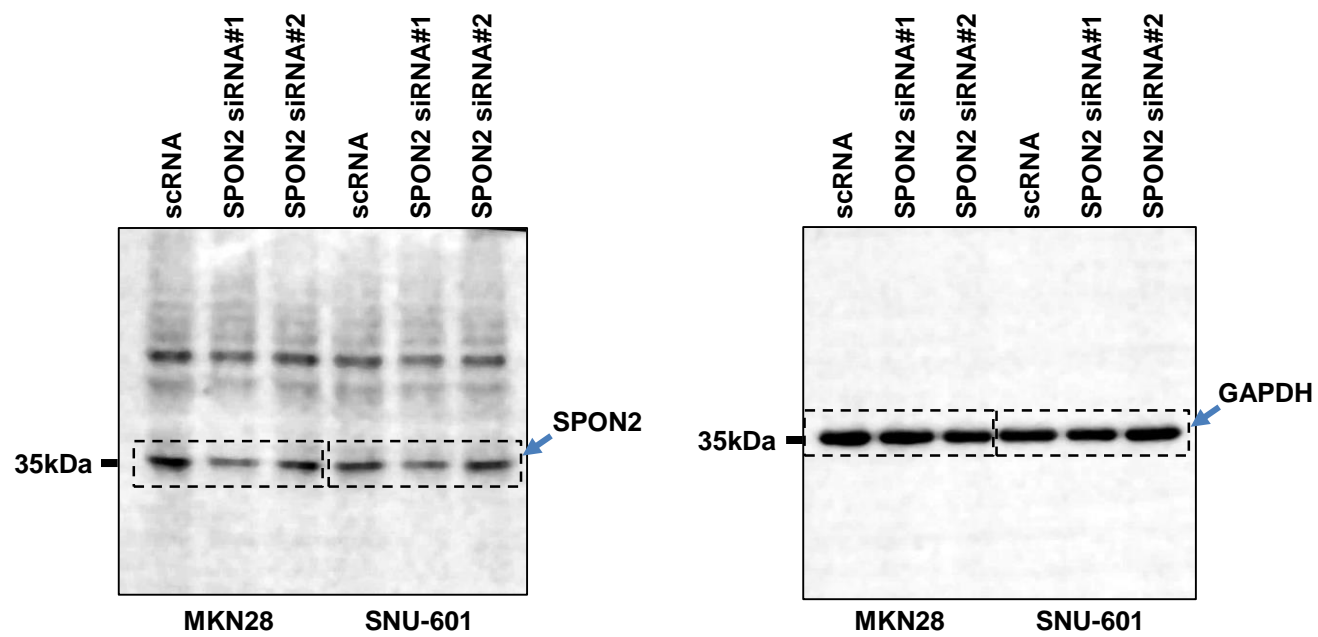

Fig. 3A

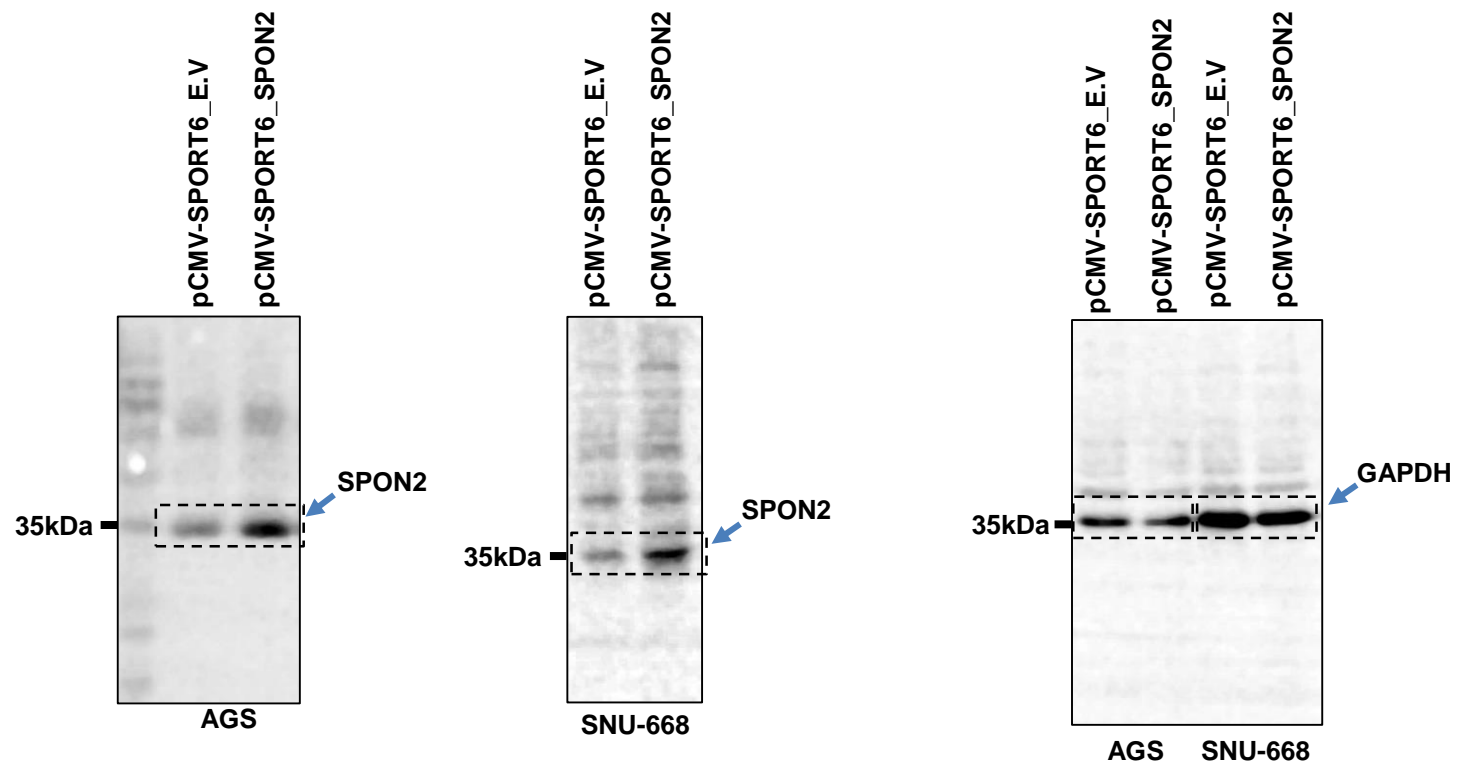

Fig. 4A

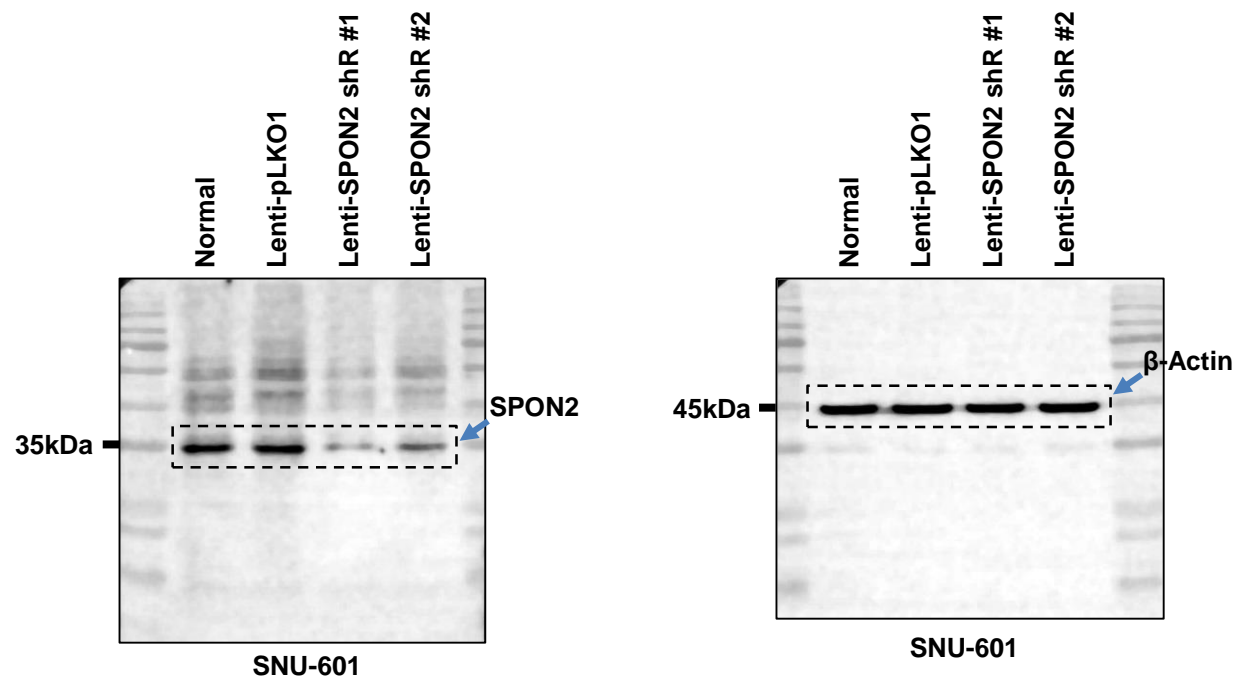

Fig. 5D

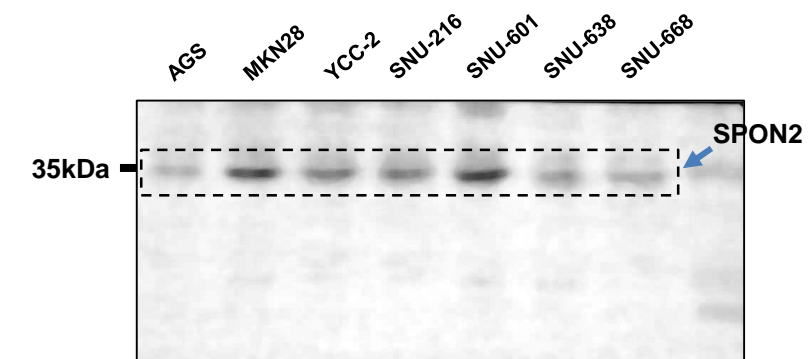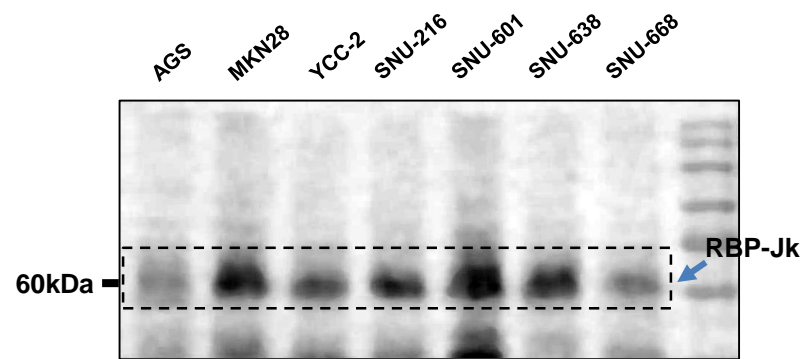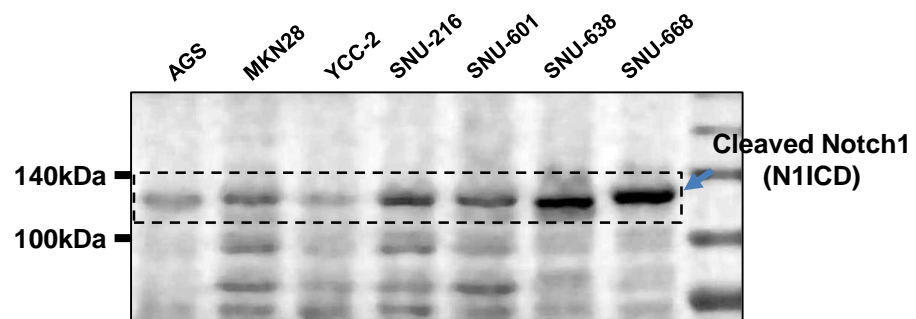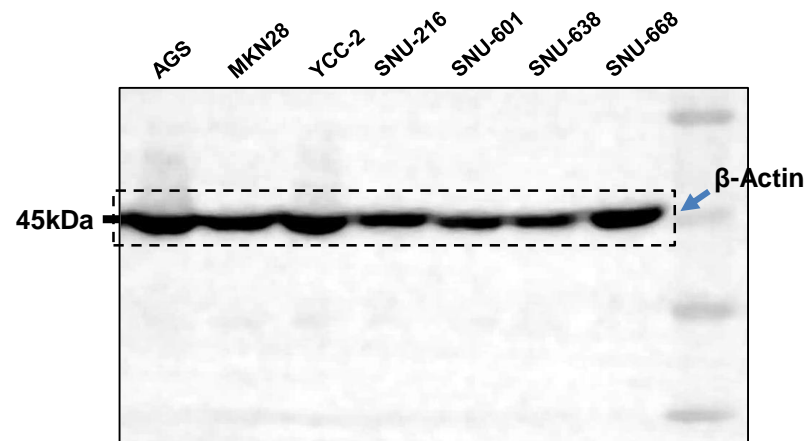

Fig. 5F

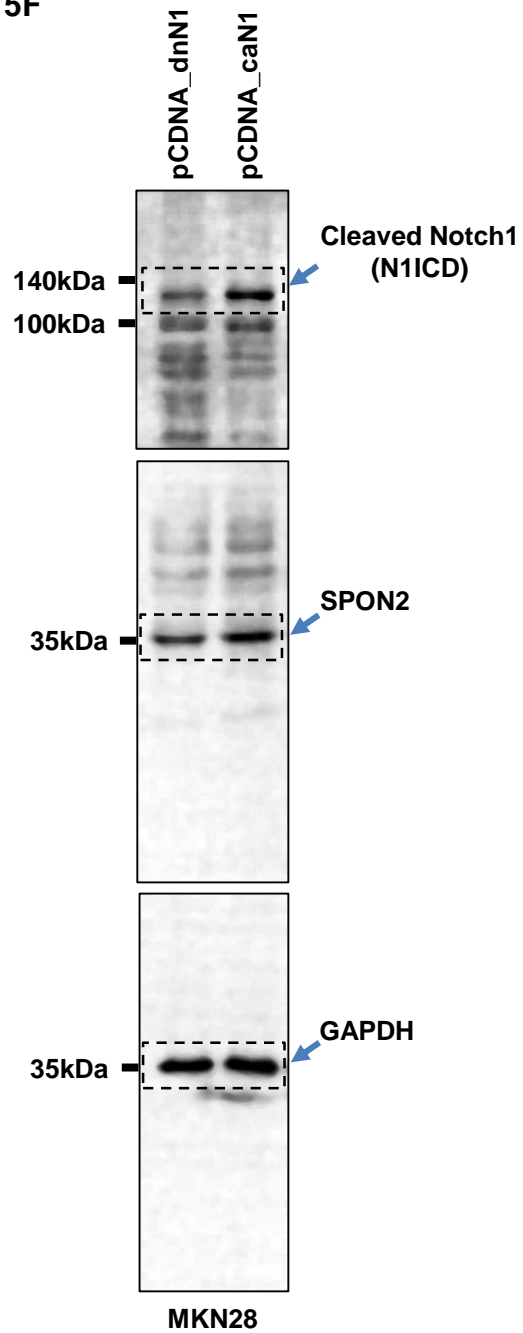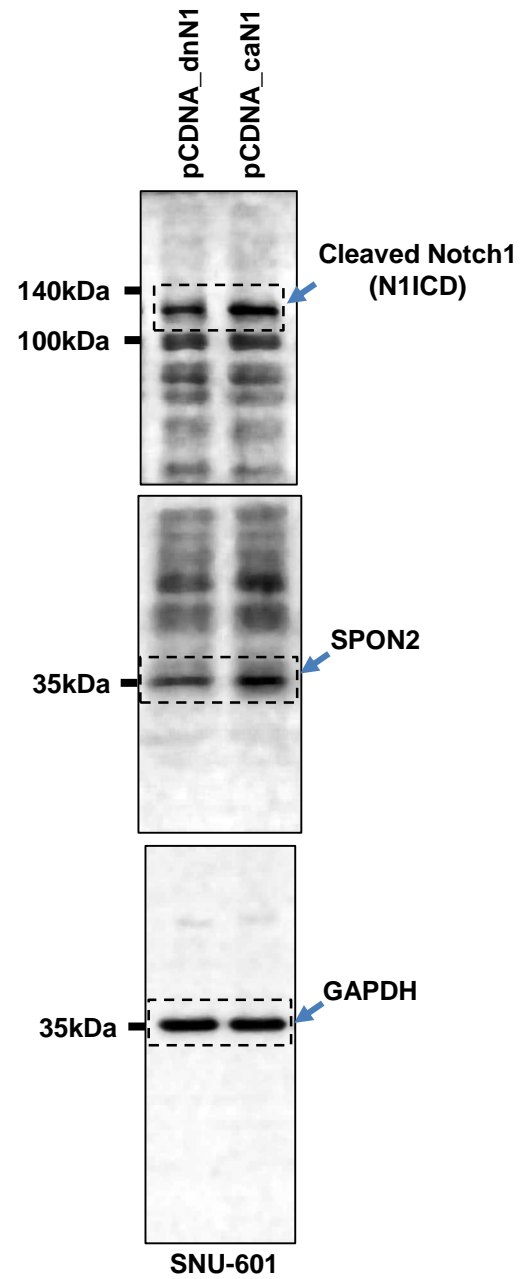

Fig. 6A

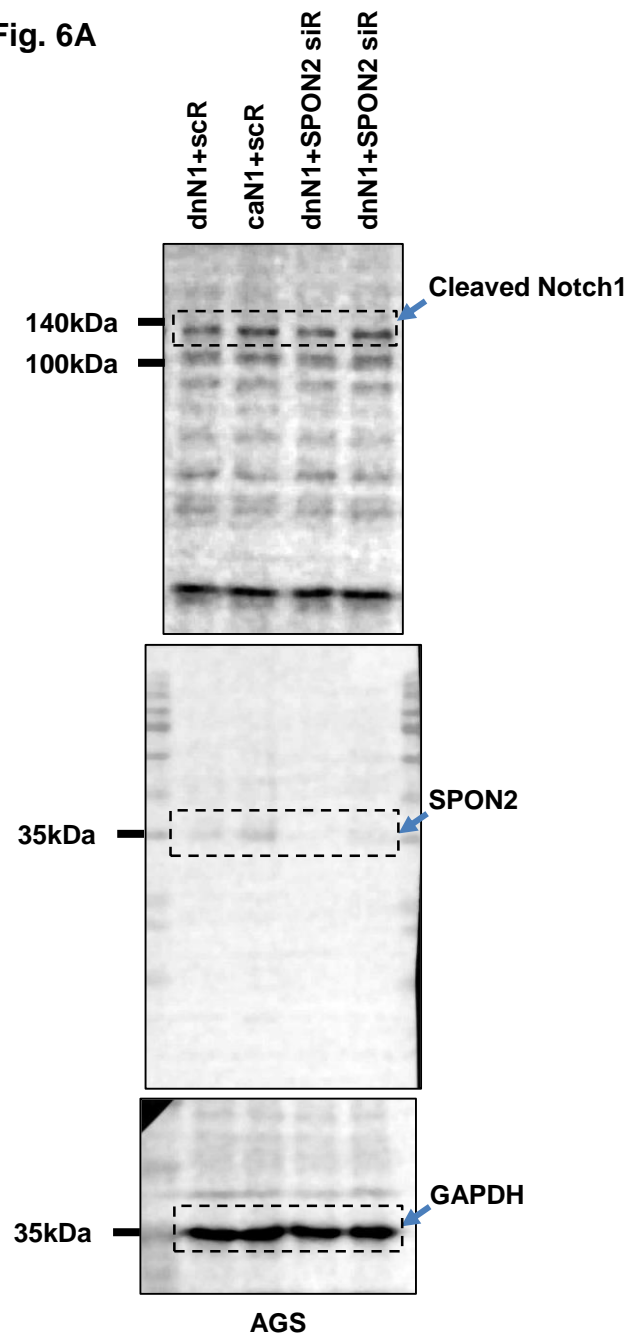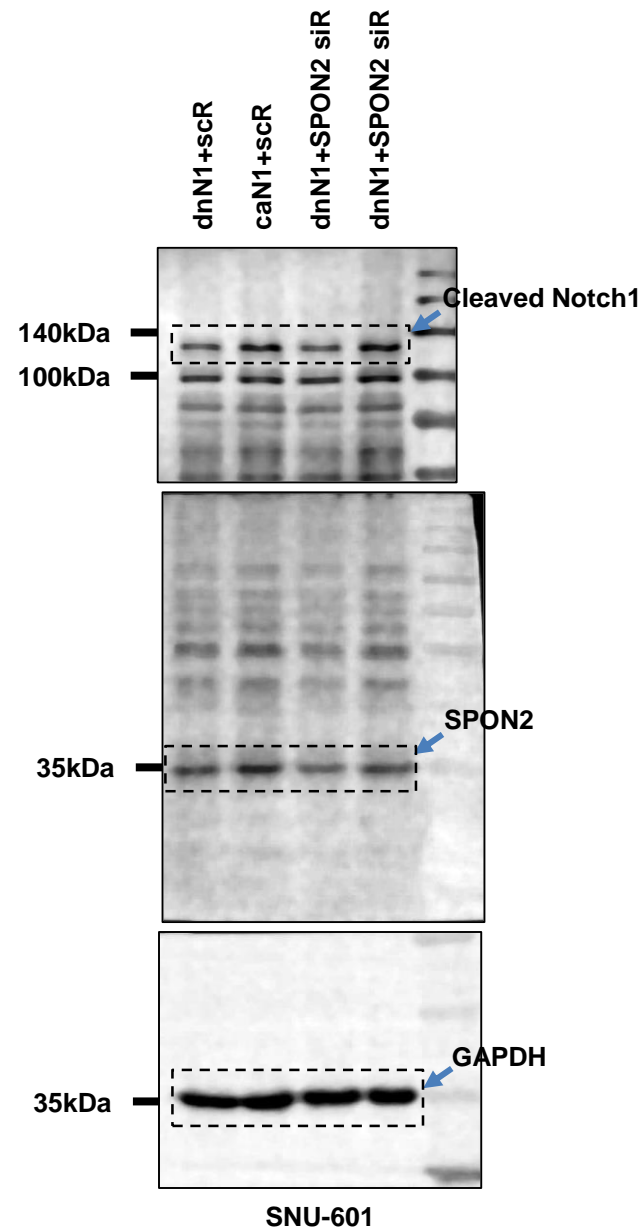

Fig. 7B

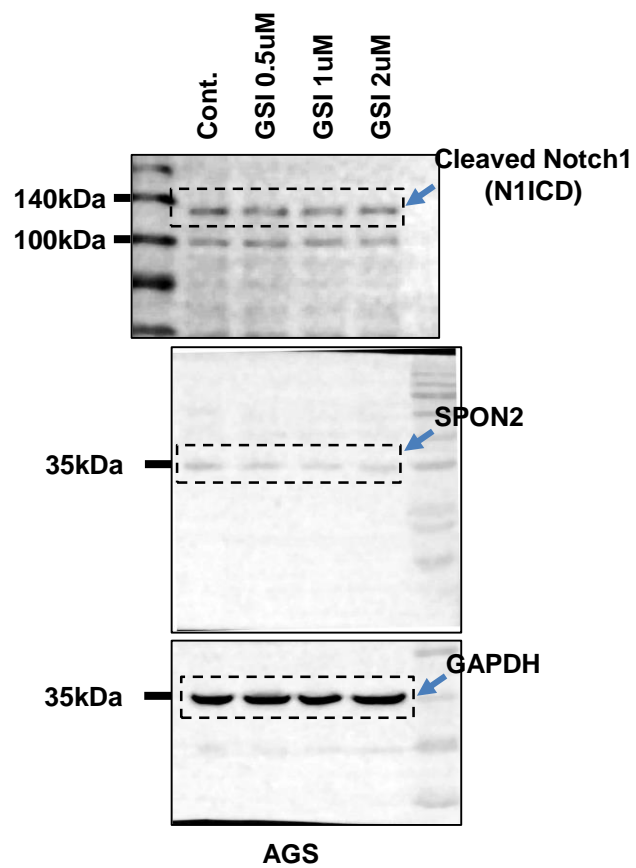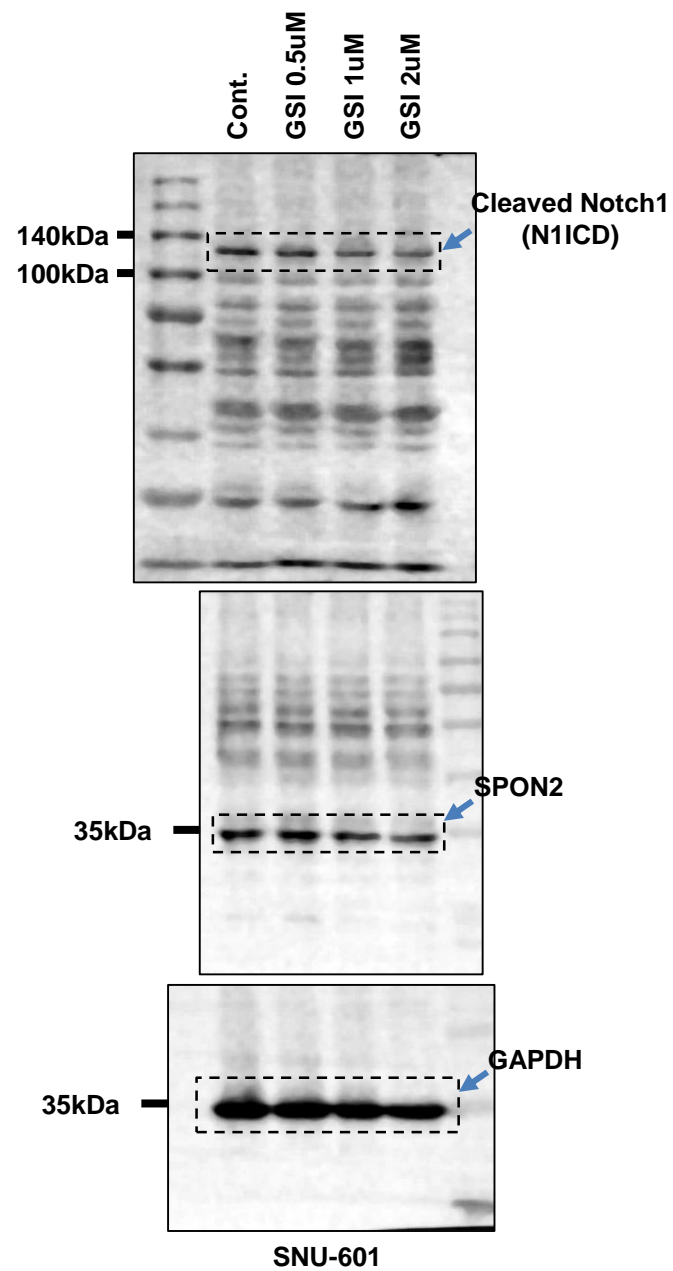

Supplement: Supplementary file 1 [file cancers-12-01439-s001.zip › cancers-803439_Western_Blot.pdf]
